# 10 Hz repetitive transcranial magnetic stimulation (rTMS) may improve cognitive function: An exploratory study of schizophrenia patients with auditory hallucinations

**DOI:** 10.1016/j.heliyon.2023.e19912

**Published:** 2023-09-09

**Authors:** Jiankai Mao, Kaili Fan, Yaoyao Zhang, Na Wen, Xinyu Fang, Xiangming Ye, Yi Chen

**Affiliations:** a Tongde Hospital of Zhejiang Province, Hangzhou, Zhejiang, PR China; b Wenzhou Seventh People's Hospital, Wenzhou, Zhejiang, PR China; c The Affiliated Kangning Hospital of Wenzhou Medical University Zhejiang Provincial Clinical Research Center for Mental Disorder, Wenzhou, Zhejiang, PR China; d The Affiliated Brain Hospital of Nanjing Medical University, Nanjing, PR China; e Zhejiang Provincial People's Hospital, Hangzhou, Zhejiang, PR China

**Keywords:** Schizophrenia, Cognitive function, Immediate memory, Auditory hallucinations, Repetitive transcranial magnetic stimulation

## Abstract

**Objectives:**

Cognitive impairment in schizophrenia patients with auditory hallucinations is more prominent compared to those without. Our study aimed to investigate the cognitive improvement effects of 10 Hz repetitive transcranial magnetic stimulation (rTMS) over the left dorsolateral prefrontal cortex (DLPFC) in schizophrenia with auditory hallucinations.

**Methods:**

A total of 60 schizophrenic patients with auditory hallucinations in this study were randomly assigned to sham or active group. Both groups received 10 Hz or sham rTMS targeted in left DLPFC for 20 sessions. The Positive and Negative Syndrome Scale (PANSS), the Auditory Hallucination Rating Scale (AHRS), the Repeatable Battery for the Assessment of Neuropsychological Status (RBANS), and the Udvalg for Kliniske Under-sogelser (UKU) side effect scale were used to measure psychiatric symptoms, auditory hallucinations, cognition, and side reaction, respectively.

**Results:**

Our results indicated that the active group experienced greater improvements in RBANS-total score (*P* = 0.043) and immediate memory subscale score (*P* = 0.001). Additionally, the PANSS-total score, negative and positive subscale score were obviously lower in the active group compared to the sham group (all *P* < 0.050). Furthermore, our study found that the improvement of RBANS-total score was positively associated with the decline of positive factor score, and the improvement of language score in RBANS was positively associated with the reduction in PANSS-total scale, negative and positive subscale score in the real stimulation group (all *P* < 0.050).

**Conclusion:**

Our results demonstrated that a four-week intervention of 10 Hz rTMS over the left DLPFC can improve cognition (particularly immediate memory) among schizophrenia patients with auditory hallucinations. Future studies with larger sample size are needful to verify our preliminary findings.

## 1 Introduction

Schizophrenia is a severe mental disorder that about 1% population in the worldwide were affected [1]. The lifetime prevalence of this disorder in China is nearly 0.7%, and the prognosis of schizophrenia patients is poor [2]. Clinically, schizophrenia mainly characterized by positive symptoms (i.e., hallucinations, delusions, behavior disorder, etc.) [3], negative symptoms (i.e., affective flattening or apathy, anhedonia, impaired attention, and social withdrawal, etc.), and cognitive impairment [4]. The conceptualization of psychotic diseases changed from that of a neurodegenerative disorder to that of a neurodevelopmental disorder [5]. According to the findings of a comprehensive meta-analytic review indicate that cognitive impairment precede the onset of psychosis, experiencing progressive deterioration, especially in later life [6]. Evidence points out that the neurocognitive impairment profile observed in patients with psychiatric disorders is similar although in different degrees [7]. Psychotic patients exhibit deficits in various cognitive functions, including immediate and delayed memory, working memory, language, motor skills, processing speed etc [8].

With the advent of chlorpromazine in the 1950s, antipsychotics began to become the dominant interventions for schizophrenia patients [9]. Evidence suggests that antipsychotics are effective at improving patients' positive symptoms, but have limited efficacy against cognitive impairment [10]. Some studies have even reported that some psychotropic medications can have detrimental effects on cognitive function [11]. A large body of literature has demonstrated that cognitive deficits are closely associated with daily function, contributing to disturbances in the social function of patients [12]. In addition, antipsychotic drugs usually accompanied by severe adverse reactions which leads to the reduction of drug compliance [[13], [14], [15]]. Moreover, the recurrence rate of the disease is relatively high, even under stable antipsychotic treatment [16]. Hence, it is highly necessary to develop newly treatment means to improve patients’ cognitive function and alleviate the psychiatric symptoms.

In recent years, some non-pharmacological interventions have been booming, such as dietary polyphenols [17], acetyl*-*L-carnitine [18], omega-3 [19]. In addition, physical therapy has also attracted the attention of many researchers. For example, repetitive transcranial magnetic stimulation (rTMS) is also a newly treatment option for all kinds of neuropsychiatric illness, including schizophrenia [20,21]. The rTMS regulates neuronal activity in the cerebral cortex by delivering repeated, rapidly changing magnetic fields through the scalp and skull to generate local electrical currents [22]. In general, high-frequency rTMS (>1 Hz) alleviates cognitive impairment and negative symptoms by increasing cortical excitability, but low-frequency rTMS (≤1 Hz) alleviates positive symptoms, including auditory hallucinations, by suppressing cortical excitability [23,24]. In schizophrenia, the existed literature generally indicates that rTMS may help to ameliorate psychological symptoms, including persistent auditory hallucinations, negative symptoms, and cognitive impairment [[25], [26], [27]]. Previous studies have also explored the effect of rTMS on cognitive impairment in schizophrenic patients with predominant negative symptoms. For instance, Xiu et al. reported that both 10 and 20 Hz rTMS treatment produced improvement of cognitive deficit in schizophrenia with predominant negative symptoms [28]. One small randomized and controlled study reported that 10 Hz rTMS showed no help on negative symptoms, but significantly improved delayed memory at the two-week follow-up [29]. Another study showed that rTMS with 10 Hz targeted in the left DLPFC markedly improved facial affect recognition in schizophrenia [30]. However, other researchers failed to find the role of rTMS on cognition in schizophrenia patient. For instance, a randomized controlled trial from multicenter indicated that active rTMS with 10 Hz was not advantageous compared to sham stimulation group in enhancing multiple aspects of cognition in schizophrenic patients with predominant negative symptoms at three-week follow-up [31]. Collectively, a review of the literature suggested that cognitive impairments in schizophrenia patients could be benefit from the rTMS modulation on the left DLPFC [32]. Given the inconsistency in the reported findings, and the evidence of rTMS on cognitive function in schizophrenia is still not sufficient, more studies are necessary to explore the potential benefits of rTMS for improving cognition in schizophrenia.

It is worth noting that most studies have focused on the efficacy of rTMS on cognition in schizophrenia with predominant negative symptoms. Little research has examined the effect of rTMS on cognitive function in patients accompanied by auditory hallucinations. Interestingly, substantial evidences suggest that patients with schizophrenia accompanied by auditory hallucinations have more obvious impairment in cognition compared to those without auditory hallucinations. For instance, Waters et al. reported that almost 90% of patients currently experiencing auditory hallucinations exhibited deficits in various cognitive functions, including memory, while only one-third of patients without hallucinations exhibited comparable deficits [33].

Thus, in this study, we selected from a population that was more likely to show considerable cognitive impairments, and aimed to explore whether high-frequency rTMS targeted in the left DLPFC ameliorates cognitive impairment in schizophrenia patients accompanied by auditory hallucinations. It was hypothesized that the cognitive impairments in these patients would benefit from 10 Hz rTMS over the DLPFC.

## 2 Methods

### 2.1 Subjects

All participants were recruited from December 2018 to December 2019 in Wenzhou Kangning Hospital. We used the Structured Clinical Interview for the Diagnostic and Statistical Manual of Mental Disorders, fourth edition (DSM-IV-TR) to diagnose schizophrenia patients. The following were the inclusion criteria: (1) Han Chinese, (2) aged between 18 and 60 years, (3) right-handed, (4) Auditory Hallucination Rating Scale (AHRS) > 10, and (5) with a stable antipsychotics dose over one month prior to enrolled in this trial.

Patients were excluded when met the following criteria: (1) serious somatic disease, such as gastrointestinal diseases, genitourinary disorders, cardiac vascular diseases or other serious medical condition, (2) fitted with a intracranial metal, cardiac pacemaker, or head injury or epilepsy history, (3) underwent electroconvulsive therapy (ECT) or rTMS within three months prior to study enrollment, (4) patient received psychotherapy, and (5) had substance abuse or dependence history, including alcohol and nicotine, and patients who used alcohol and nicotine within the half year before enrollment, (6) pregnant or breastfeeding,

The clinical research proposal was approved by the ethics committee at Wenzhou Kangning Hospital (approval number: KNLL-2018111001). After being fully explained, each participant and/or their legal guardian(s) signed an informed consent form prior to the study initiation. Patients included in this study receive rTMS therapy free of charge.

### 2.2 Study design

A total of 60 patients were enrolled consecutively in this preliminary double-blind, randomized, controlled pilot trial. Each participant was randomly allocated to the sham or active rTMS (10 Hz) group. An unbiased third party used computer-generated simple randomization to allocate the participants to either the active or sham group, with 30 patients in each group. Only the rTMS technician who conducted the study protocol knowing the grouping the patients; both the patients and the clinical staff were blinded to the therapy being given. We used the Positive and Negative Syndrome Scale (PANSS), The AHRS, the Repeatable Battery for the Assessment of Neuropsychological Status (RBANS), and the Udvalg for Kliniske Under-sogelser (UKU) side effect scale to assess the psychiatric symptoms, auditory hallucinations, cognition, and side reaction among these patients.

### 2.3 Intervention

A YRDCCY-I stimulator (Yiruide Medical Equipment New Technology Co., Ltd., Wuhan, China) with a figure-eight-shaped coil was used to perform rTMS. Before each rTMS session, the rest motor threshold (rMT) was selected by single-pulse stimulation targeted in the left primary motor cortex; The minimal intensity required to elicit motor-evoked potentials of 0.05 mV amplitude in 5 out of 10 consecutive trials was defined as rMT [34]. The left DLPFC stimulus site was determined as the point 5.5 cm anterior to the scalp position at which the rMT had been selected. In the active group, rTMS over the left DLPFC occurred at a power of 110% of the MT for 4 s, with 26-s intervals, totaling 1600 pulses every session reach 20 min daily. The coil of rTMS was tangent to the skull plane, with its middle was aligned with the stimulation position. The sham group had the identical procedures with the active group except for an angled 90° off of the coil over the head in the sham group [35].

Over four weeks, each patient underwent 20 sessions of sham or active rTMS with 10 Hz targeted in the left DLPFC. The patients were awake and in a comfortable siting position during rTMS treatment.

### 2.4 Clinical assessments

The RBANS was used for cognitive function evaluation among schizophrenia patients, which consists of five domains: immediate memory, delayed memory, language, attention, and visuospatial/constructional. It has strong reliability and validity in the Chinese population and performs well in cognitive assessments among schizophrenia [36]. Higher RBANS scores indicate better cognitive performance in general [37]. The AHRS was applied to evaluate auditory hallucinations of schizophrenia patients, which contains seven items: number of distinct speaking voices, frequency, vividness, perceived loudness, attentional salience, hallucinations length, as well as distress degree [38]. In general, higher AHRS scores indicate a greater symptom burden. The PANSS [1,39] was used to evaluate the psychopathological symptoms among patients. with higher scores indicate more symptom burden. The PANSS five-factor model was used in our study, which contain: excited factor, depression factor, cognitive factor, negative factor and positive factor [40]. The UKU side effect rating scale was used to measure the side reaction in schizophrenia. It includes 48 items that cover psychological, neurologic, autonomic, and other negative consequences [41].

Assessments were completed by highly trained clinicians before and after intervention. To ensure the consistency and reliability of ratings, all assessments were implemented by two professionally trained psychiatrists. The test-retest reliabilities of all scales were greater than 0.8 after training [42].

### 2.5 Outcomes

The primary outcome was the improvement of cognition (RBANS score) after rTMS intervention, while the secondary outcome was the changes of the psychopathological symptoms (AHRS and PANSS score) in schizophrenia patients. Moreover, the association between improved cognitive function and reduced clinical symptoms was investigated.

### 2.6 Data analysis

SPSS version 22.0 was used for data analyses. The categorical variables were described as proportions, and the continuous data were displayed as means ± standard deviations. The chi-squared and the student's t-test and the chi-squared test were used for demographic and clinical characteristics comparisons at baseline. After age, sex, duration of illness, education level, daily antipsychotic dose, and baseline scores were controlled as covariant, analysis of covariance (ANCOVA) was applied to examine the post-therapeutic (four weeks) data. In addition, all analyses of the post-intervention data used repeated measurements. Then we used a mixed-effects model for repeated measures analysis to evaluate the intervention and time effects regarding to the changes of psychopathological symptoms and cognition in the recruited patients. The Bonferroni was used for multiple comparisons. The association between cognition and psychopathological symptoms was investigated using partial correlation analysis. *P* < 0.05 (two-tailed) was considered significant in all models.

## 3 Results

### 3.1 Demographic and clinical data

**Fig. 1:**
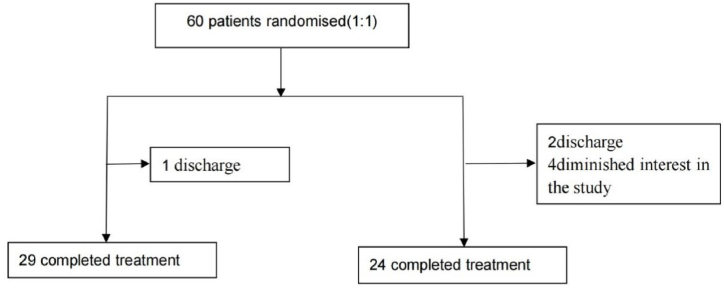
The study flow chart. Note: rTMS, repetitive transcranial magnetic stimulation.

Thirty patients were allocated in 10 Hz rTMS group and 30 in sham rTMS group. Eventually, twenty-nine patients completed the four-week active treatment, whereas twenty-four patients completed the four-week sham treatment. Four patients dropped out of the study because of early discharge from hospital, while three dropped out early because they refused to continue treatment (Fig. 1).

Throughout the study period, patients’ antipsychotic dosages remained constant. The antipsychotic medications taken by the patients during the study period included clozapine (12 in active group and 14 in sham group), risperidone (10 vs. 8), aripiprazole (9 vs. 7), quetiapine (7 vs. 3), olanzapine (3 vs. 5), amisulpride (2 vs. 2), and ziprasidone (3 vs. 0). As shown in Table 1, our results indicated no statistical differences in the chlorpromazine-equivalents doses and antipsychotic type between active and sham groups (all *P* > 0.05). Furthermore, we found no differences in age, sex, education level, disease causes, age of onset, RBANS, AHRS, and PANSS scores between groups at baseline.

**Table 1:** Baseline demographics and clinical characteristics in the active and sham group.

	10 Hz(n = 29)	Sham(n = 24)	X^2^or T	*P*-value
Age(years)	38.17 ± 7.60	38.25 ± 9.16	-0.03	0.97
Sex(female)	16(55%)	10(42%)	0.96	0.33
Education(years)	8.55 ± 3.64	8.66 ± 2.94	-0.13	0.90
Age of onset(years)	23.17 ± 6.99	24.54 ± 8.65	-0.64	0.53
Duration of illness	15.00 ± 7.07	13.71 ± 6.64	0.68	0.50
DAD(mg)	418.21 ± 283.83	517.07 ± 319.54	-1.07	0.18
clozapine	12	14		
olanzapine	3	5		
quetiapine	7	3		
risperidone	10	8		
aripiprazole	9	7		
amisulpride	2	2		
ziprasidone	3	0		
RBANS total score	58.37 ± 12.15	56.95 ± 9.59	0.47	0.64
Immediate memory	51.65 ± 14.13	53.79 ± 13.15	-0.57	0.57
Visuospatial/constructional	69.83 ± 14.98	70.13 ± 10.74	-0.08	0.94
Language	68.34 ± 15.45	64.37 ± 13.25	0.99	0.33
Attention	71.31 ± 15.89	71.46 ± 15.42	-0.03	0.97
Delayed memory	63.10 ± 20.02	55.83 ± 15.53	1.45	0.14
AHRS total score	25.62 ± 4.16	24.87 ± 3.64	0.69	0.50
PANSS total score	107.27 ± 12.92	109.00 ± 12.27	-0.49	0.62
Positive factor	13.48 ± 2.89	14.92 ± 4.04	-1.50	0.14
Negative factor	23.58 ± 4.37	23.75 ± 3.31	-0.15	0.88
Cognitive factor	12.62 ± 2.35	13.04 ± 1.83	-0.72	0.48
Depression factor	9.38 ± 2.13	8.58 ± 1.55	1.52	0.14
Excited factor	11.62 ± 3.53	12.58 ± 2.78	-1.09	0.28

Note: DAD, daily antipsychotic dose (mg) (chlorpromazine equivalent); RBANS, repeatable battery for the assessment of neuropsychological status; AHRS, Auditory Hallucination Rating Scale; PANSS, Positive and Negative Syndrome Scale.

### 3.2 Effect of rTMS treatment on cognitive function

**Fig. 2:**
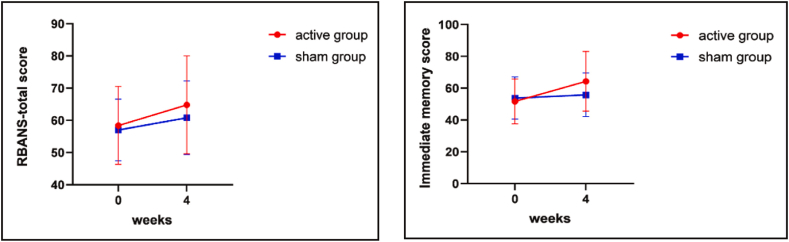
Changes in the RBANS-total scores and immediate memory score between active rTMS and sham group at baseline and endpoint (4th week).

Table 2 provides the ANCOVA results for the RBANS scale and its subscales after rTMS intervention. After sex, age, education, illness causes, chlorpromazine equivalents doses, and baseline score were controlled, the immediate memory score (F_(1,51)_ = 12.7, *P* = 0.001, *P*_Bonferroni-corrected_ < 0.050) and RBANS-total score (F_(1,51)_ = 4.3, *P* = 0.043, *P*_Bonferroni-corrected_ < 0.050) in the rTMS group were substantially higher compared to the sham group. The repeated-measures ANOVA demonstrated a notable interaction eﬀect (group × time: F = 8.4, df = 1,51, *P* < 0.01) and time eﬀect (F = 37.2, df = 1,51, *P* < 0.001) and an interaction eﬀect (group × time: F = 8.4, df = 1,51, *P* < 0.010),but no group eﬀect (F = 0.69, df = 1,51, *P* = 0.409) on immediate memory. Our results indicated only a signiﬁcant time eﬀect on the RBANS total score (F = 37.24, df = 1,51, *P* < 0.001), visuospatial (F = 6.09, df = 1,51, *P* < 0.05), language(F = 5.89, df = 1,51, *P* < 0.05), attention (F = 11.18 df = 1,51, *P* < 0.01) and delayed memory (F = 13.69, df = 1,51, p < 0.010), after age, education level, illness causes, chlorpromazine equivalents doses were controlled (see Table 4). Moreover, the changes in the immediate memory (F = 0.14, df = 1, 51, *P* = 0.001, *P*_Bonferroni-corrected_ < 0.050) and RBANS-total score (F = 1.72, df = 1, 51, *P* = 0.043，*P*_Bonferroni-corrected_ < 0.050) in the rTMS group from baseline to post intervention were more remarkable compared to the sham group (Fig. 2), with sex, age, education, illness causes, drug dose (chlorpromazine equivalents), and baseline score as covariates. However, there were no significant improvements in the other subscales of the RBANS (all *P* > 0.050).

**Table 2:** Comparison of the results of the RBANS-total score and subscale score between the two groups at the end of the week 4 measure using ANCOVA.

	10 Hz(n = 29)	sham(n = 24)	*P*-value^a^	Effect size η2
RBANS total score	64.79 ± 15.15	60.75 ± 11.44	0.043*	0.53
Immediate memory	64.28 ± 18.73	55.83 ± 13.66	0.001*	0.94
Visuospatial/constructional	73.83 ± 16.14	74.33 ± 13.82	0.786	0.06
Language	71.52 ± 15.05	69.25 ± 13.25	0.086	0.05
Attention	75.62 ± 15.76	77.29 ± 16.39	0.687	0.07
Delayed memory	69.83 ± 22.31	62.88 ± 17.59	0.689	0.07

Note: ^a^*P*-value, by comparisons of between-group differences using analysis of covariance, the baseline characteristics were considered as covariates. RBANS, repeatable battery for the assessment of neuropsychological status. *P < 0.05.

**Table 3:** Comparison of AHRS total score, PANSS-total score and subscale score between the two groups at the end of the week 4 measure using ANCOVA.

	10 Hz(n = 29)	sham(n = 24)	*P*-value^a^	Effect size η2
AHRS total score	25.06 ± 4.65	24.58 ± 3.53	0.551	0.09
PANSS total score	88.86 ± 11.59	95.38 ± 12.20	0.020*	0.66
Positive factor	10.10 ± 2.40	12.25 ± 3.89	0.048*	0.51
Negative factor	20.52 ± 3.41	21.67 ± 2.41	0.027*	0.59
Cognitive factor	11.21 ± 2.04	12.08 ± 1.93	0.096	0.25
Depression factor	8.14 ± 1.75	7.13 ± 1.83	0.196	0.25
Excited factor	8.06 ± 2.74	9.37 ± 3.21	0.332	0.16

Note: ^*a*^*P*-value, by comparisons of between-group differences using analysis of covariance, the baseline characteristics were considered as covariates. AHRS, Auditory Hallucination Rating Scale; PANSS, Positive and Negative Syndrome Scale. *P < 0.05.

**Table 4:** Primary and secondary outcome measures at the beginning and the end of 4weeks of rTMS treatment.

	Baseline (*n* = 53)		After treatment (*n* = 53)		Group	Time	Group × Time
	Sham rTMS (n = 24)	Active rTMS (n = 29)	Sham rTMS (n = 24)	Active rTMS (n = 29)	*F* (*p-*value)	*F* (*p-*value)	*F* (*p-*value)
RBANS total score	56.96 ± 9.59	58.38 ± 12.15	60.75 ± 11.44	64.79 ± 15.15	0.678(0.414)	37.242(0.000)	2.459(0.123)
Immediate memory	53.79 ± 13.16	51.66 ± 14.13	55.83 ± 13.66	64.28 ± 18.73	0.692(0.409)	16.226（0.000)	8.447(0.005**)
Visuospatial	70.13 ± 10.75	69.83 ± 14.98	74.33 ± 13.82	73.83 ± 16.14	0.013（0.91）	6.093(0.017）	0.004（0.95）
Language	64.38 ± 13.25	68.35 ± 15.45	69.25 ± 13.25	71.52 ± 15.05	0.748（0.391）	5.889（0.019）	0.264（0.61）
Attention	71.46 ± 15.42	71.31 ± 15.89	77.29 ± 16.39	75.62 ± 15.76	0.049（0.826）	11.177（0.002）	0.252（0.618）
Delayed memory	55.83 ± 15.53	63.10 ± 20.03	62.88 ± 17.59	69.83 ± 22.31	2.041（0.159）	13.693（0.001）	0.007（0.932）
AHRS total score	24.88 ± 3.64	25.62 ± 4.16	24 .58 ± 3.54	25.07 ± 4.66	0.318(0.575)	2.705(0.106)	0.257(0.614)
PANSS total score	109.00 ± 12.28	107.28 ± 12.93	95.38 ± 12.20	88.86 ± 11.59	1.839(0.181)	115.6(0.000)	2.582(0.114)
Positive factor	14.92 ± 4.04	13.48 ± 2.90	12.25 ± 3.89	10.10 ± 2.40	4.785（0.033*）	54.87（0.000）	0.762（0.387）
Negative factor	23.75 ± 3.31	23.59 ± 4.37	21.67 ± 2.41	20.52 ± 3.41	0.551（0.461）	44.93（0.000）	1.644（0.206）
Cognitive factor	13.04 ± 1.83	12.62 ± 2.35	12.08 ± 1.93	11.21 ± 2.04	1.49（0.228）	33.65（0.000）	1.24（0.271）
Depression factor	8.58 ± 1.56	9.38 ± 2.13	7.13 ± 1.83	8.14 ± 1.75	4.489（0.039*)	24.253(0.000)	0.157(0.694)
Excited factor	12.58 ± 2.78	11.62 ± 3.52	9.38 ± 3.21	8.07 ± 2.74	2.464(0.123)	55.735(0.000)	0.144(0.706)

RBANS, repeatable battery for the assessment of neuropsychological status. AHRS, Auditory Hallucination Rating Scale; PANSS, Positive and Negative Syndrome Scale. *P < 0.05, **P < 0.01.

### 3.3 Effect of rTMS treatment on psychotic symptoms

**Fig. 3:**
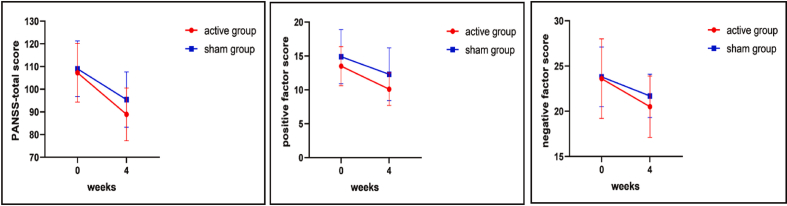
Changes in the PANSS-total score, positive factor score and negative factor score between active rTMS and sham group at baseline and endpoint (4th week).

Table 3 provides the ANOVA results for the AHRS and PANSS scales after the rTMS intervention. After sex, age, education, illness causes, chlorpromazine equivalents doses, and baseline score were controlled, we found no significant difference in the AHRS score between these two group (*P* > 0.050). The active group had a substantially lower positive factor score (F_(1,51)_ = 4.1, *P* = 0.048, *P*_Bonferroni-corrected_ < 0.050), negative factor score (F_(1,51)_ = 5.2, *P* = 0.027, *P*_Bonferroni-corrected_ < 0.050), and PANSS-total score (F_(1,51)_ = 5.9, *P* = 0.020, *P*_Bonferroni-corrected_ < 0.050) than the sham group after above covariates were controlled (Table 3). Our repeated-measures ANOVA further indicated a notable group (F = 4.79, df = 1,51, *P* = 0.033) and time eﬀects (F = 54.9, df = 1,51, *P* < 0.001), but no interaction eﬀect (F = 0.76, df = 1,51, P = 0.387) on positive factor. For depression factor, there were also a signiﬁcant group (F = 4.79, df = 1,51, *P* = 0.039) and time eﬀect (F = 24.3, df = 1,51, *P* < 0.001), but no signiﬁcant interaction eﬀect (F = 0.16, df = 1,51, P = 0.694). our results reported only a signiﬁcant time eﬀect on the PANSS total score (F = 115.6, df = 1,51, *P* < 0.001), negative factor (F = 44.9, df = 1,51, *P* < 0.001), cognitive factor (F = 33.7 df = 1,51, *P* < 0.001), and excited factor (F = 55.74, df = 1,51, *P* < 0.001) (Table 4). Furthermore, the rTMS group exhibited significant reductions in the positive factor score (F = 0.35, df = 1,51, *P* = 0.04, *P*_Bonferroni-corrected_ < 0.05), negative factor score (F = 0.16, df = 1,51, *P* = 0.027，*P*_Bonferroni-corrected_ < 0.05) and PANSS-total score (F = 0.05, df = 1,51, *P* = 0.02，*P*_Bonferroni-corrected_ < 0.05) after interevention (Fig. 3), even when sex, age, education, illness causes, chlorpromazine equivalents doses, and baseline score were controlled. However, no significant improvements in the other subscales were found (all *P* > 0.050).

### 3.4 Correlation analyses in the 10 Hz rTMS group

After age, education, chlorpromazine equivalents doses, and baseline score were controlled, our results revealed that the increases in the RBANS-total score from baseline to week four were markedly correlated with the reductions in PANSS-positive factor score (r = 0.231, df = 21, *P* = 0.013) in the active group. In addition, we found significant correlations between the increases in the language score on the RBANS and the following parameters: the reductions in the PANSS total score (r = 0.456, df = 21, *P* = 0.029), positive factor score (r = 0.417, df = 21, *P* = 0.048), and negative factor score (r = 0.446, df = 21, *P* = 0.033) in the active group.

### 3.5 Safety assessment

After the four-week intervention, neither group experienced any severe side effects (severe side effects were defined as those that interfered with the patient's function based on the patient's and physician's evaluations). Two patients in active group had minor side effects (one with nervousness and one with increased salivation), and 3 patients in sham group had minor side effects (one with drowsiness and two with increased salivation). All other patients tolerated rTMS well.

## 4 Discussion

For all we know, our study was the first to discuss and analyze the efficacy of 10 Hz rTMS on cognitive function in schizophrenia patients accompanied by auditory hallucinations. The principal findings of the current study were: (1) rTMS significantly improved RBANS-total and immediate memory scores in schizophrenia patients accompanied by auditory hallucinations, indicating that rTMS could promote the recovery of cognition, especially immediate memory, in schizophrenia patients; (2) rTMS significantly reduced PANSS total scores, negative factors and positive factor scores (to some degree) in these patients; and (3) the improvement in cognitive function was associated with reduced psychopathological symptoms after active rTMS intervention.

In this clinical trial, patients who received the 10 Hz rTMS therapy obviously increases in immediate memory score and RBANS-total score compared to the control group, suggesting that rTMS produces clinical improvements in cognitive function among schizophrenia patients with auditory hallucinations. Other researchers have also reported positive effects of rTMS on cognitive function in schizophrenic patients. For instance, Wolwer et al. reported that rTMS with 10 Hz applied to the left DLPFC markedly promote facial affect recognition among patients with schizophrenia [30]. A meta-analysis demonstrated that rTMS treatment with high-frequency positioned on the left DLPFC had a substantial promotion on working memory in schizophrenia patients, compared to sham stimulation [43]. Existed evidence has also suggested that rTMS in high-frequency can improve memory impairment in schizophrenia [44]. The current results provide further evidence that rTMS in high-frequency over the left DLPFC can effectively promote cognitive function, even for schizophrenia patients with auditory hallucinations. Interestingly, the cognitive factor in PANSS did not show a significant change after 10 Hz rTMS intervention. The possible explanation may be due to the different sensitivity of these two assessment tools for cognition evaluation. As we know, the RBANS has a good practicability in cognitive assessments among Chinese patients with schizophrenia, while the PANSS mainly applied to evaluate the degree of positive and negative symptoms in those patients. In addition, the cognitive factors, including three items in PANSS (G11: Poor attention, N5: Difficulty in abstract thinking, and P2: Conceptual disintegration), only reflects a fraction of cognitive function and as a preliminary judgment of cognitive impairment in schizophrenia patients. Hence, its evaluation validity and reliability on cognition in schizophrenia patients is limited. The present findings highlighted the importance of selecting evaluation tools, and future studies are warrant to further confirm this finding.

The precise mechanisms with regard to the effect of rTMS on cognition in schizophrenia are still unclear, which may be related to the following factors. First, rTMS can regulate the release of neurotransmitters and induce neuronal long-term potentiation or depression, thereby altering synaptic plasticity [45]. Early studies suggested that the unbalanced dopamine (DA) metabolism in schizophrenia patients impairs the function of the left DLPFC and then causing abnormalities in functional connectivity with other brain regions, thus resulting in cognitive impairment. Animal experiments have proven that high-frequency rTMS can induce prefrontal cortical modulation of DA release [46], thereby contributing to the improvement of cognitive function deficits in schizophrenic patients. BDNF is known to involve in the pathological mechanism of cognitive function, and its peripheral levels can serve as a index for the assessment of cognition in schizophrenia [47]. High-frequency rTMS could increase the affinity of BDNF for TrkB and then improve BDNF-TrkB signaling in rats [48]. In addition to neuroplastic phenomena, rTMS can also regulate the hypothalamic-pituitary-gonadal (HPG) system, leading to an over release of hormones such as estradiol and prolactin. Studies suggest that the HPG axis, which plays multiple roles in brain development and function, affects cognitive function [49,50]. Patients with cognitive impairment often have decreased glucose metabolism in cental system, and rTMS with high-frequency can contribute to increased glucose metabolism in brain regions [51,52]. Furthermore, studies have shown that rTMS with high-frequency can cause more active cerebral blood flow and improve the energy metabolism of brain cells, thus improving cognitive function [53,54]. Moreover, studies have reported that patients with schizophrenia have significantγoscillations deficits, which are related to the higher-order cognitive function [55]. rTMS with high-frequency may promote cognitive function among schizophrenia through modulatingγoscillatory activity in the brain [56]. In summary, rTMS has a complex series of effects, including altering synaptic plasticity, influencing the function of the neuroendocrine system, increasing cerebral blood flow to improve metabolism in certain brain areas, and modulate the oscillation of brain waves; all of these effects could potentially responsible for the alleviation of cognitive deficits among patients with schizophrenia. Further investigations are required to uncover the precise mechanisms with regard to the effects of 10 Hz rTMS on cognition function in schizophrenia.

Additionally, the efficacy of 10 Hz rTMS on psychopathological symptoms in schizophrenia accompanied by auditory hallucinations was also examined in this trial. The results revealed that four weeks of 10 Hz rTMS significantly reduced the negative subscale and PANSS-total subscale score in the active group, and somewhat reduced the positive factor score. This indicates that rTMS improved the negative symptoms and some of the positive symptoms in these subjects. This finding is similar to previous studies. For example, Quan et al. found that six-week therapy with 10 Hz rTMS decreased negative symptoms compared to the control group and that this effect lasted until the 24-week follow-up examination [57]. Novak et al. [58] investigated the efficacy of a two-week 20 Hz rTMS intervention on the left DLPFC and found significant improvements in schizophrenia patients’ positive symptoms after eight weeks. The mechanism of the rTMS treatment on psychiatric symptoms could be related to the following factors. First, the low activity in the DLPFC may be associated with the negative symptoms among schizophrenic patients [59], and high-frequency rTMS may stimulate the cerebral cortex and the neurons and then improve negative symptoms in those patients [60]. Second, studies suggest that positive symptoms arise due to deficiencies in parts of the DA pathway in the brain, such as a primary decrease in D1 receptors in the prefrontal lobe and a compensatory over release of DA from other pathways such as the midbrain limbic system DA pathway. rTMS induces DA release in the prefrontal cortex [61], resulting in a feedback inhibitory effect on DA release in the limbic system of the midbrain. This may be why rTMS improves some of the negative symptoms while also improving some of the positive symptoms.

In here, we also investigate the effect of rTMS on auditory hallucination among these patients, but failed to draw a meaningful conclusion. Kimura et al. also reported that rTMS (20 Hz) stimulation of the left temporoparietal cortex (TPC) produced no obvious difference in auditory hallucinations between active and sham group after four weeks treatment [62]. However, other studies have demonstrated a meaningful treatment effect of rTMS on auditory hallucinations in schizophrenia. One meta-analysis reported that rTMS with low-frequency significantly reduced auditory hallucinations in schizophrenia [63]. In this study, the lack of significant improvement in auditory hallucinations may be related to the stimulus frequency of rTMS used in this study. Previous studies have suggested that rTMS with low-frequency (≤1 Hz) over the TPC can obviously ameliorate auditory hallucinations [64]. Therefore, the different protocol used in our study may be the explanation that no significant effect on auditory hallucinations was observed. In addition, the lack of effect may be attributed to the relatively long causes of disease of the patients in this study; most of the patients here had been repeatedly hospitalized [65,66]. Furthermore, the short observation period of the current study may not have allowed us to observe an effect. Therefore, further larger samples studies are necessary to explore the best treatment modality for rTMS to improve auditory hallucinations in schizophrenia. It is important to note that, in this study, although rTMS did not significantly alleviate auditory hallucinations, the greater attrition rate in sham group relative to the active group might indirectly reflect a better subjective experience of the individuals with active rTMS treatment.

Our results also suggested that the cognitive improvement after rTMS intervention may be associated with the relieve of psychopathological symptoms in these patients. This is consistent with many previous studies [28]. Numerous literatures have indicated that deficits in cognition shared common pathological mechanisms with the psychopathological symptoms in schizophrenia [67]. In schizophrenia, abnormal levels of neurotransmitters that interact with BDNF have been linked to cognitive impairments and psychotic symptoms. However, this association and the exact mechanism behind it are not fully understood and deserve further investigation.

The following are the limitations of the present study. First, the rTMS location was determined by manual measurement. Although the “5 cm rule” is standard practice, literatures have indicated that neuro-navigation is more precise for determinizing the stimulus site [68]. Second, only in the coil placement angle of the sham group is different from that of the active stimulus group, which may damage the blindness, compromise the placebo and affect the results to some extent. Finally, the small sample size, which might impair the statistical power. Nonetheless, no study, by now, have examined the efficacy of rTMS over the left DLPFC on cognitive deficits among schizophrenia accompanied by auditory hallucinations. Thus, this study was a first attempt and preliminary investigation. The results of this study should be replicated in larger samples. Fourth, we did not conduct the post hoc blindness test, such as calculating the Bang's index, to check if our blind method has been successfully implementation. Fifth, our study without long-term tracking of the patients, hence, future longer-term follow-up studies are required. Moreover, our study only selected one protocol of the rTMS stimulation, the best protocol of rTMS is needed to be explored to verify our findings in the furture [69]. Finally, all subjects maintained their drug regimens throughout the trial, it is possible that the patients' psychotropic medications may have altered their cognitive performance and clinical symptoms.

Overall, this exploratory study with 10 Hz rTMS protocol for a total of 20 treatments targeted in the left DLPFC showed an improvement of cognitive function, particularly immediate memory, and negative symptoms, positive symptoms in our sample of schizophrenia patients with auditory hallucinations. However, our study only with limited number of patients. In addition, this study was not able to determine how long the potential improvement lasted. Further research to address these limitations is required.

## Author contribution statement

Jiankai Mao, Kaili Fan, Yi Chen: Conceived and designed the experiments; Performed the experiments; Analyzed and interpreted the data; Contributed reagents, materials, analysis tools or data; Wrote the paper. Yaoyao Zhang, Na Wen: Performed the experiments; Analyzed and interpreted the data; Contributed reagents, materials, analysis tools or data; Wrote the paper. Xinyu Fang, Xiangming Ye: Conceived and designed the experiments; Analyzed and interpreted the data; Wrote the paper.

## Data availability statement

The data that has been used is confidential.

## Ethics approval and consent to participate

The ethics committee of Affiliated Kangning Hospital approved this study (KNLL-2018111001). All methods were carried out in compliance with the Declaration of Helsinki and the relevant guidelines. After being fully explained, each participant and/or their legal guardian(s) provided written consent before the study began.

## Funding

The Wenzhou Science and Technology Program provided funding for this study (S20190026).

## Declaration of competing interest

The authors declare that they have no known competing financial interests or personal relationships that could have appeared to influence the work reported in this paper.
